# Ozone Pollution, Perceived Support at Home, and Asthma Symptom Severity in the Adolescent Sample of the California Health Interview Survey

**DOI:** 10.1007/s12529-022-10103-8

**Published:** 2022-06-02

**Authors:** Theodore F. Robles, Sunhye Bai, Ying-Ying Meng

**Affiliations:** 1 Department of Psychology, University of California, 1285 Psychology Building, Box 951563, Los Angeles, CA 90095-1563 USA; 2 Department of Human Development and Family Studies, The Pennsylvania State University, 216 Health and Human Development Building, University Park, PA 16802 USA; 3 Center for Health Policy Research, UCLA Fielding School of Public Health, 10960 Wilshire Blvd, Suite 1550, Box 957143, Los Angeles, CA 90095-7143 USA

**Keywords:** Family, Social support, Childhood adversity, Air pollution, Asthma, Ozone

## Abstract

**Background:**

Outdoor air pollution, including ozone (O_3_) pollution, and childhood family environments may interact and impact asthma exacerbations in children. Previous epidemiology studies have primarily focused on stress in the home, rather than support, and whether psychosocial factors modify the association between pollution and health outcomes, rather than whether pollution exposure modifies associations between psychosocial factors and health outcomes.

**Methods:**

Data from the cross-sectional 2003 representative, population-based California Health Interview Survey were linked with air quality monitoring data on O_3_ pollution from the California Air Resources Board. Adolescents (*N* = 209) ages 12–17 who reported an asthma diagnosis and lived within 5 mi of the nearest air monitoring station had linked O_3_ data for a 12-month period preceding the survey interview date. Adolescents reported perceived available support from an adult at home and frequency of asthma symptoms.

**Results:**

In unadjusted models, for adolescents living in high O_3_ pollution regions, greater perceived support was related to lower asthma symptom frequency. Follow-up analyses suggested that the most plausible interpretation of the interaction was that O_3_ exposure modified the association between perceived support and symptom frequency. O_3_ × perceived support interactions were not statistically significant after adjusting for covariates.

**Conclusions:**

These data provide preliminary evidence that the association between the lack of support in the home environment and worse asthma symptoms may be stronger in areas with higher O_3_ exposure. Future work may benefit from incorporating personal pollution exposure assessments, comprehensive family environment assessments, and longitudinal follow-up of asthma exacerbations over time.

## Introduction

Environmental exposures that can impact asthma severity range from the composition of the air that children breathe [1–3] to the quality of the home family environment [4–7]. Environmental and social exposures likely interact, which may explain why the association between outdoor air pollution and asthma exacerbations in children and adolescents has a small and heterogeneous effect size (ORs between 1.03 and 1.05) [2, 3]. Contributors to heterogeneity include the wide range, mix, and sources of pollutants, including ozone (O_3_,) nitrogen oxides (NO_x_), and particulate matter and individual “host factors” (gender, age, race/ethnicity, etc.) [1]. Unpacking such heterogeneity is critical for developing personalized asthma control strategies [8] that can mitigate the harmful effects of different exposures.

Innate immune-mediated inflammation may explain how pollution and the home family environment interact to impact asthma symptoms [4–6]. In the airways, pollutants can increase oxidative stress, stimulate innate immune responses, and enhance acquired immune responses to inhaled allergens implicated in allergic asthma, thereby worsening asthma symptoms [1]. Moreover, growing up in a family characterized by high conflict and low warmth may prime the immune system to vigorously respond to danger signals such as cellular damage caused by environmental pollutants [4–6, 9, 10]. At the same time, innate immune responses may influence social cognition and behavior [11] via neural pathways involved in social information processing [12–14] which may then have downstream impacts on peripheral inflammation [15]. While biologically plausible, epidemiological data are needed to provide evidence of the phenomenon and inform the direction of the interaction. That is, does the social environment modify the association between pollution exposure and asthma outcomes, or does pollution exposure modify the association between social environments and asthma outcomes?

Two studies in the Los Angeles region, which ranks near or at the top of the list of most polluted cities in the USA [16], primarily focused on stressful family environments magnifying the association between pollution exposure and respiratory function [17, 18]. In the Children’s Health Study (*N* = 1,399), the association between higher NO and NO_x_ exposure at home and lower forced expiratory volume was larger for children whose parents reported high perceived stress [17]. In the Los Angeles Family and Neighborhood Survey (*N* = 551), greater NO_x_ exposure was related to worse lung function for children without a father at home [18]. In both studies, psychosocial factors did not show a main effect association with lung function and measures of the family psychosocial environment were limited — neither included well-validated child reports of conflict or support in the family environment. Moreover, neither study focused on children with diagnosed asthma nor reported whether pollution exposure modified the association between psychosocial factors and lung function.

Thus far, the only study to report whether the association between psychosocial factors and lung function in children with asthma might be modified by pollution exposure comes from a sample of 73 children and adolescents with asthma [4]. At lower relative levels of nitrogen dioxide (NO_2_) exposure, greater chronic stress exposure was related to worse lung function. At higher relative levels of NO_2_ exposure, greater chronic stress exposure was related to better lung function. Importantly, the study was conducted in Vancouver, British Columbia, Canada, which has a lower air pollution burden compared to many urban areas around the world, including in the USA [19].

The aforementioned studies focused on stress and conflict, but the balance of conflict and support in the home plays a central role in how family relationships impact children’s health [20]. Support in the home, defined as interpersonal interest, involvement, and assistance [21] may protect children from the detrimental effects of environmental exposures on health through pathways that include greater medication adherence [22] and lower innate immune-mediated inflammation [23]. However, to date no studies have examined whether support in the home environment may interact with pollution exposure to impact asthma symptoms.

Thus, using cross-sectional data from the 2003 biennial, population-based California Health Interview Survey (CHIS), this study examined whether support from an adult in the home interacted with pollution exposure to predict asthma symptom frequency in adolescents with asthma. In addition to including questions about perceived social support in the home environment [24], respondents’ residential addresses in the 2003 CHIS were linked to air pollution data from the nearest California Air Resources Board (CARB) air monitoring station.

We focused on O_3_, which is formed through chemical reactions involving NOx, volatile organic compounds, and solar radiation and importantly has a more uniform spatial distribution compared to other pollutants [2, 25]. In addition, ambient outdoor O_3_ exposure levels may be more accurately estimated for adolescents who spend more time outdoors compared to very young children [25]. Data from air monitoring stations, while limited compared to exposure modeling [26], may provide proof of concept for examining interactions between pollution exposure and the family psychosocial environment, even after controlling for family socioeconomic status as a background stressor. We explored two possibilities: (1) greater perceived support may buffer against the negative impact of O_3_ exposure on asthma outcomes, such that in adolescents reporting higher perceived support, we would observe no association between O_3_ exposure and asthma symptom severity, or (2) greater O_3_ exposure may increase sensitivity to the social environment, such that for adolescents living in regions characterized by high air pollution, greater perceived support at home would be related to lower asthma symptoms and exacerbations.

## Methods

### Participants and Procedures

The 2003 CHIS Adolescent sample (*N* = 4010 between ages 12 and 17 [27]), was obtained through a random-digit dial telephone survey of households from August 2003 to February 2004, within 41 geographic strata representing all counties in California, with oversampling of underrepresented minority households. In households with children, one child (age < 12) and/or one adolescent was randomly selected and interviewed after obtaining parent/guardian consent [details in 24]. Interviews lasted an average of 21.5 min [24]. The overall household response rate was 57.3% for the adolescent interview and 83% in households where parents gave permission for the adolescent interview. For this study, respondents (*N* = 476) were included if they responded “yes” to the question “Has a doctor ever told you or your parents that you have asthma?” and “yes” to one of the following two questions: “Do you still have asthma?” or “During the past 12 months, have you had an episode of asthma or an asthma attack?” Participant data was linked to data from the nearest CARB air monitoring station within 20 mi of the respondent’s residential address (Esri ArcGIS, Redlands, CA) or nearest cross-streets or zip codes if residential address was not available (4.2% and 11.9% of the sample, respectively). Because the spatial variability of ozone increases considerably after 5 mi from air monitoring sites, similar to prior work with these data [25], we further restricted analyses to participants who lived within 3 and 5 mi of the nearest air monitoring station (*N* = 112 and 209, respectively; participant characteristics are shown in Table 1). Secondary data analyses of CHIS data were approved by UCLA’s Institutional Review Board.

**Table 1:** Descriptive statistics for demographics, asthma outcomes, pollution exposure, and perceived support by samples ≤ 3 and ≤ 5 mi from air monitoring station

Variable	≤ 3 mi sample (*N* = 112)	≤ 5 mi sample (*N* = 209)
Self-reported demographics		
Age	14.6 [14.2, 15.0]	14.5 [14.1, 14.8]
Sex (% female)	51.8	51.1
Ethnicity (%)		
Latino	24.5	29.1
Native American	3.9	4.5
Asian/Asian American	6.0	8.2
African American	22.7	16.5
White	39.3	34.5
Pacific Islander/Other	3.7	7.2
Poverty level (%)		
0–100	20.5	21.6
101–200	15.8	17.5
201–300	15.4	16.6
> 301	48.3	44.4
Health insurance		
Yes	94.6	96.2
No	5.4	3.8
Asthma outcomes		
Symptom frequency, 1–5 scale	2.46 [2.28, 2.64]	2.48 [2.33, 2.63]
Not at all	8.0	10.5
Less than every month	51.8	50.2
% intermittent	59.8	60.7
Every month	19.6	21.5
Every week	18.8	15.3
Every day	1.8	2.4
% persistent	40.2	39.2
% currently taking daily prescription control medication	33.5	37.6
Episode or attack, past 12 months	33.1	34.8
ER/urgent care visit for asthma, past 12 months	9.5	6.4
O_3_ pollution		
Mean concentration (ppb)	40.7 [39, 43]	41.7 [40, 43]
Unweighted *SD*	8.4	8.2
Unweighted range	23–59	23–59
Perceived available adult support in the home		
Mean	3.65 [3.5, 3.8]	3.66 [3.6, 3.7]
Unweighted *SD*	0.40	0.41
Unweighted range	2.2–4	2.17–4

Means and 95% *CIs* [in brackets] derived using jackknife replicate weights method to obtain correct variance estimates accounting for two-stage geographically stratified random-digit dial sample design

### Measures

#### Ozone Pollution

Annual averages of daily 8-h maximums provided by CARB were computed for the 12-month period preceding the respondent’s CHIS interview date. At least 15 daily values/month had to be available for a given monthly average. Annual averages were then computed for participants who had 12 monthly values available. For regression analyses, annual average O_3_ was scaled by 10 ppb (descriptive statistics are reported as in 1 ppb units), a common practice in epidemiological studies of air pollution.

#### Perceived Support at Home

Adolescents were asked to endorse how true (1 = not at all true, 4 = very much true) the following six statements were about “your home or the adults with whom you live” and whether there was a parent or some other adult in the home who “cares about your schoolwork,” “listens to you,” “talks with you about your problems,” “wants you to do your best,” “believes you will be a success,” and “notices your bad moods.” The first five items were derived from a measure of supportive relationships at home that is part of an optional module of the California *Healthy Kids Survey*^1^ assessing environmental assets that contribute to resilience [28]. Responses were averaged across items (Cronbach’s α = 0.80). The perceived support from an adult at home score was negatively skewed, as most adolescents reported support in the “pretty much true” to “very true” range (Table 1). These items have only appeared on the 2003 and 2016 CHIS Adolescent surveys.

#### Asthma Outcomes

Adolescents responded 1 = not at all, 2 = less than once a month, 3 = monthly, 4 = every week, or 5 = every day, to a single item about past 12-month frequency of symptoms*:* “how often have you had asthma symptoms such as coughing, wheezing, shortness of breath, tightness or phlegm?” The primary outcome was symptom frequency analyzed as a continuous dependent variable. As a secondary outcome, respondents were defined as having persistent asthma (with 4 = daily or 5 = weekly symptoms) vs. intermittent asthma (with 3 = monthly, 2 = less than monthly, or 1 = no symptoms)^2^ [30, 31]. Secondary asthma-related outcomes included two yes or no items: “have you had an episode of asthma or an asthma attack” and “have you visited the emergency room (ER) or urgent care for asthma” in the past 12 months.

#### Covariates

Adjusted models included the following covariates: adolescent-reported age (in years, centered); gender (0 = male); race/ethnicity including White, African American, Latino and all others; and current daily prescription asthma medication use (0 = none); parent-reported health insurance status (0 = currently insured); and calculated poverty level based on parent-reported household income and size (301% federal poverty level and above, 201–300%, 101–200%, and 0–100%) (more information available at [30]).

### Data Analysis

The two-stage geographically stratified random-digit dial sample design of CHIS required proper weighting and variance calculation using jackknife replicate weights to reduce selection and non-respondent biases [24]. Weighting adjustments used a raking method that accounted for demographics, geographic variables, household composition, and socioeconomic variables. These sample weights were applied in all data analyses. Any missing values were replaced by random selection from the distribution of respondents and hot deck imputation without replacement. All analyses described below were repeated with adolescents living ≤ 3 mi and ≤ 5 mi from the nearest air monitoring station. The ≤ 3 mi sample, while it has a smaller sample size, should have more accurate estimates of O_3_ for a given individual. The ≤ 5 mi sample has a larger sample size; depending on the radius of adolescents’ daily activity areas, this might still provide a valid O_3_ measurement for a given individual.

Our primary dependent variable of interest was asthma symptom frequency, which was analyzed using linear regressions, and significant statistical interactions between support and O_3_ were interpreted by computing simple slopes and Johnson–Neyman regions of significance [32]. Secondary dependent variables modelled with logistic regressions were likelihood of having intermittent vs. persistent asthma over the past year, likelihood of having an asthma episode/attack, and an ER or urgent care visit for asthma in the past 12 months. Unless otherwise specified, all regression coefficients (*B*’s) are unstandardized, and values between brackets indicate 95% *CIs*.

## Results

### Descriptive Statistics for Asthma Outcomes

Adolescent-reported symptom frequency corresponded to between less than once a month to every month (Table 1). Most adolescents reported intermittent symptoms (59.8–60.7%). Just over one-third of the sample reported taking daily prescription controller medication, and one-third of the sample reported experiencing an episode or attack in the past 12 months. The prevalence of ER/urgent care visits in the past 12 months was < 10%.

### Associations Among Demographics, Asthma Outcomes, Ozone Pollution, and Support

Higher O_3_ was related to higher perceived support, ≤ 3 mi *B* = 0.47 [0.10, 0.84], *p* = 0.013, *R*^*2*^ = 0.07, and ≤ 5 mi *B* = 0.40 [0.12, 0.69], *p* = 0.006, *R*^2^ = 0.04. O_3_ was not related to asthma symptom frequency at ≤ 3 mi, *B* = − 0.13 [− 0.37, 0.11], *p* = 0.29, or ≤ 5 mi, *B* = − 0.06 [− 0.26, 0.13], *p* = 0.51.

To present associations among demographics, perceived support, and asthma outcomes, we focus on the ≤ 5 mi sample. Perceived support was not related to age, race/ethnicity, daily medication use, or having insurance. Adolescents from the lowest poverty level (0–99%) reported less perceived support compared to adolescents from the highest poverty level (> 300%), *B* = − 0.25 [− 0.46, − 0.04], *p* < 0.05; perceived support did not differ between the three highest poverty level groups. Older children reported greater symptom frequency, *B* = 0.10 [0.004, 0.20], *p* = 0.042. However, symptom frequency and medication use were not related to sex, race/ethnicity, poverty level, or having insurance. Reporting using daily asthma medication use was related to higher symptom frequency, *B* = 0.67 [0.31, 1.04], *p* < 0.001. The likelihood of having an asthma attack or visiting the ER/urgent care in the past year was not related to age, sex, poverty level, or insurance status. White adolescents were more likely to report an asthma attack in the past year, *OR* = 2.81 [1.01, 7.83], *p* = 0.048, compared to Latino adolescents (the reference group, which did not differ from African American or American Indian/Asian/Pacific Islander/other categories). Children in the American Indian/Asian/Pacific Islander/other category were less likely to visit the ER, *OR* = 0.23 [0.08, 0.68], *p* = 0.009, compared to Latino adolescents (who did not differ from African American or White adolescents).

### Primary Analyses: Support × Ozone Pollution Interactions

**Fig. 1:**
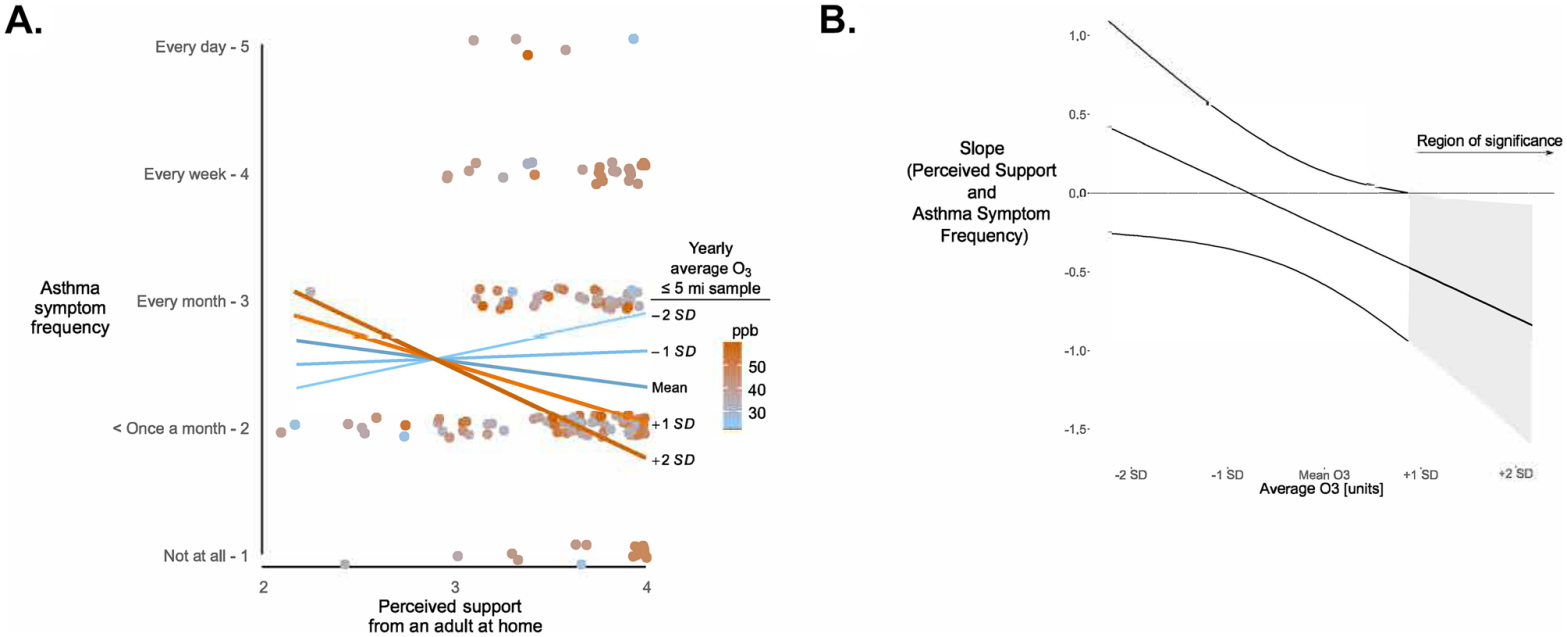
**A** Self-reported asthma symptom frequency as a function of yearly average O_3_ (− 2 *SD* to + 2 *SD*) and perceived support from an adult at home in the ≤ 5 mi sample. Points are jittered vertically and horizontally to minimize overlap. Orange lines and dots represent higher O_3_. **B** Slope of the association between perceived support from an adult at home and asthma symptom frequency, as a function of yearly average O_3_. The shaded region of significance indicates when the slope of the association between the perceived support from an adult at home and asthma symptom frequency is statistically significant, at ≥ 48.8 ppb. The unshaded region indicates the slope is not significantly different from zero at values < 48.8 ppb. The color version is available online. The figure is in grayscale in the print version

When asthma symptom frequency was treated as a continuous variable, there were no main effects of O_3_ and of perceived support on frequency of symptoms, and significant O_3_ × perceived support interactions were observed for participants living ≤ 3 and ≤ 5 mi of an air monitoring station but only in unadjusted models (top portion of Table 2). We computed two sets of Johnson–Neyman regions of significance, one where support was the moderator and the other where O_3_ exposure was the moderator. When support was the moderator, the region of significance for the association between O_3_ exposure and symptom frequency becoming statistically significant was well outside the plausible range of values for the support moderator. Thus, the most plausible interpretation of the interaction was that O_3_ exposure modified the association between perceived support and symptom frequency. Figure 1A shows that in the ≤ 5 mi sample, high perceived support at home was related to lower symptom frequency but only for adolescents living in a region with high O_3_ exposure. For example, the simple slope of the association between perceived support and symptom frequency in a low O_3_ exposure region (mean –1 *SD*) was 0.06 (*SE* = 0.21), *t* = 0.31, *p* = 0.76; by comparison, in a high O_3_ exposure region (mean + 1 *SD*), the simple slope was − 0.51 (*SE* = 0.25), *t* = -2.04, *p* = 0.045. Figure 1B shows that the slope of the association between perceived support and symptom frequency becomes statistically significant when O_3_ ≥ 48.8 ppb. However, after controlling for covariates, the O_3_ × perceived support interaction was in the same direction but no longer statistically significant (both ≤ 3 and ≤ 5 mi samples).

**Table 2:** Unadjusted and adjusted linear regressions predicting frequency of symptoms from O_3_, perceived support, and the O_3_ × perceived support interaction

*Models* Variable	Distance from air monitoring station							
	≤ 3 mi (*N* = 112)				≤ 5 mi (*N* = 209)			
	*B*	*95% CI*	*t*	*p*	*B*	*95% CI*	*t*	*p*
*Unadjusted*								
O_3_	− 0.13	− 0.36, 0.11	− 1.1	.29	− 0.06	− 0.25, 0.13	− 0.6	.54
Perceived support at home	− 0.28	− 0.68, 0.12	− 1.4	.16	− 0.22	− 0.58, 0.14	− 1.2	.22
O_3_ × perceived support	− 0.61	− 1.06, -0.17	− 2.7	.008	− 0.35	− 0.70, − 0.002	− 2.0	.049
*Adjusted*								
Age	0.08	− 0.04, 0.20	1.33	.19	0.13	0.05, 0.22	3.11	.003
Sex = female	− 0.30	− 0.58, − 0.01	− 2.03	.045	− 0.19	− 0.46, 0.08	− 1.41	.16
Ethnicity (reference: White)								
Latino	− 0.57	− 0.99, − 0.14	− 2.64	.01	− 0.39	− 0.69, − 0.10	− 2.63	.01
African American	− 0.45	− 0.87, − 0.03	− 2.15	.035	− 0.57	− 0.94, − 0.22	− 3.18	.002
Other ethnicity	− 0.44	− 0.81, − 0.08	− 2.41	.02	− 0.19	− 0.60, 0.22	− 0.93	.36
Poverty level (reference: > 300%)								
0–99	0.30	− 0.13, 0.72	1.40	.17	0.33	− 0.05, 0.72	1.72	.09
100–199	0.17	− 0.29, 0.63	0.74	.46	0.18	− 0.14, 0.50	1.11	.27
200–299	0.23	− 0.14, 0.61	1.25	.21	0.12	− 0.29, 0.54	0.58	.56
Has health insurance	− 0.33	− 0.99, 0.32	− 1.02	.31	− 0.40	− 0.94, 0.14	− 1.47	.15
Currently taking daily prescription control medication	0.71	0.37, 1.06	4.09	.00	0.80	0.50, 1.10	5.36	.00
O_3_	− 0.23	− 0.46, − 0.002	− 2.0	.048	− 0.12	− 0.29, 0.06	− 1.30	.20
Perceived support at home	− 0.08	− 0.51, 0.34	− 0.4	.70	− 0.10	− 0.51, 0.30	− 0.51	.61
O_3_ × perceived support	− 0.40	− 0.83, 0.03	− 1.87	.065	− 0.26	− 0.60, 0.09	− 1.49	.14

Jackknife replicate weights used in all data analysis. Unadjusted models *R*^*2*^: ≤ 3 mi = .07, ≤ 5 mi = .02. Adjusted models *R*^*2*^: ≤ 3 mi = .26, ≤ 5 mi = .28

**Fig. 2:**
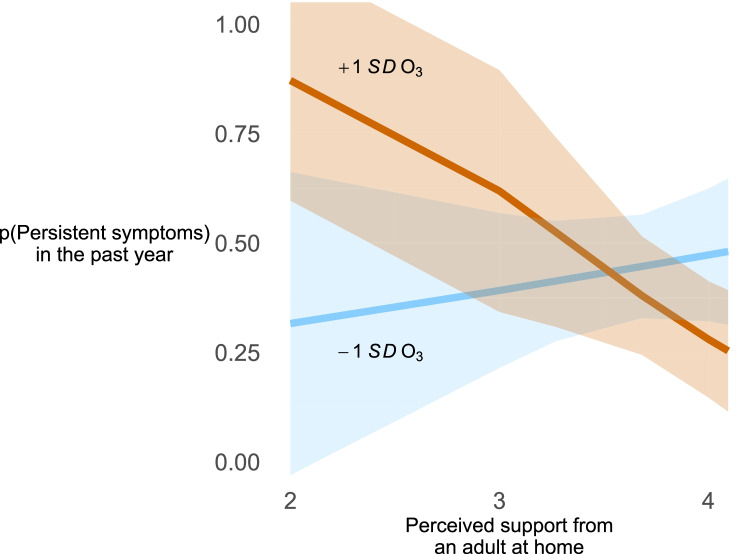
Probability of persistent asthma in the past year as a function of yearly average O_3_ (− 1 *SD* and + 1 *SD*) and perceived support from an adult at home in the ≤ 5 mi sample. Bands represent 95% *CIs* around probability estimates. The color version is available online. The figure is in grayscale in the print version

Approximately 40% of the sample reported persistent asthma symptoms (Table 1). Similar to the linear regressions, there were no main effects of O_3_ and of perceived support on likelihood of intermittent vs. persistent symptoms in unadjusted and adjusted models (Table 3). At the same time, there was a statistically significant O_3_ × perceived support interaction in unadjusted models (≤ 3 mi *B* = − 2.61 [− 4.3, − 0.92], *t* = − 3.1, *p* = 0.003; ≤ 5 mi *B* = − 1.08 [− 2.06, − 0.10], *t* = − 2.2, *p* = 0.035). Figure 2 shows that high perceived support at home was related to lower likelihood of persistent asthma symptoms but only for adolescents in a region with high O_3_ exposure. For example, in adolescents living in a high O_3_ exposure region (mean + 1 *SD*) in the ≤ 5 mi sample, the estimated probability of reporting persistent symptoms was 52.5% [30.8, 74.1] for those reporting –1 *SD* perceived support (3.27 on the 4-point scale) compared to 31.8% [15.7, 41.3] for those reporting perceived support = 4 (because of skew the mean + 1 *SD* was outside of the scale range).

**Table 3:** Logistic regression predicting persistent vs. intermittent asthma from O_3_, perceived support, and the O_3_ × perceived support interaction, adjusting for covariates

*Models* Variables	Distance from air monitoring station							
	≤ 3 mi (*N* = 112)				≤ 5 mi (*N* = 209)			
	*B*	95% *CI*	*t*	*p*	*B*	95% *CI*	*t*	*p*
*Unadjusted*								
O_3_	− 0.33	− 0.99, 0.33	− 0.98	0.33	− 0.17	− 0.62, 0.29	− 0.74	.46
Perceived support at home	− 0.99	− 2.07, 0.10	− 1.81	.07	− 0.55	− 1.41, 0.31	− 1.27	.21
O_3_ × perceived support	− 2.61	− 4.30, − 0.92	− 3.08	.003	− 1.08	− 2.06, − 0.10	− 2.18	.03
*Adjusted*								
Age	0.11	− 0.31, 0.53	0.51	.61	0.37	0.05, 0.68	2.29	.03
Sex = female	− 0.28	− 1.21, 0.65	− 0.60	.55	− 0.3	− 0.89, 0.83	− 0.07	.94
Ethnicity (reference: White)								
Latino	− 0.87	− 2.45, 0.71	− 1.10	.28	− 0.96	− 2.00, 0.08	− 1.83	.07
African American	− 0.72	− 2.19, 0.75	− 0.98	.33	− 1.57	− 2.97, − 0.16	− 2.22	.03
Other ethnicity	− 0.91	− 2.43, 0.60	− 1.21	.23	− 0.27	− 1.63, 1.08	− 0.40	.69
Poverty level (reference: > 300%)								
0–99	0.33	− 1.31, 1.96	0.40	.69	1.08	− 0.34, 2.49	1.51	.13
100–199	0.04	− 1.40, 1.19	0.06	.96	0.40	− 0.68, 1.47	0.73	.47
200–299	1.16	− 0.25, 2.58	1.64	.11	0.61	− 0.71, 1.92	0.92	.36
Has health insurance	− 2.30	− 5.42, 0.82	− 1.47	.15	− 2.08	− 4.44, 0.28	− 1.75	.08
Currently taking daily prescription control medication	1.71	0.58, 2.84	3.02	.003	1.91	1.07, 2.76	4.51	.00
O_3_	− 0.56	− 1.36, 0.24	− 1.38	.17	− 0.33	− 0.96, 0.29	− 1.06	.29
Perceived support at home	− 0.85	− 2.21, 0.52	− 1.24	.22	− 0.38	− 1.44, 0.68	− 0.71	.48
O_3_ × perceived support	− 2.49	− 4.47, − 0.51	− 2.5	.015	− 1.08	− 2.14, − 0.02	− 2.0	.045

Jackknife replicate weights used in all data analysis to accommodate the two-state stratified sample design of CHIS. Adjusted models including the following covariates: self-reported age, self-reported sex, ethnicity (White, Latino, African American, Other), poverty level (categories in Table 1), having health insurance, taking medications to control asthma

In unadjusted and adjusted logistic regressions with the ≤ 3 and ≤ 5 mi samples, there were no main effects of O_3_ concentration or perceived support on the likelihood of an episode or attack, or ER/urgent care visits, or statistically significant O_3_ × perceived support interactions (all *p*’s > 0.09).

## Discussion

As adolescents become more independent, knowing that adults/parents are available when needed takes on increasing importance [20]. In unadjusted models, consistent with prior theories [5, 6, 9, 10], among adolescents with 12-month average O_3_ exposure greater than 48.8 ppb, reporting more perceived support at home was related to lower asthma symptom frequency, and reporting less perceived support at home was related to greater risk of persistent asthma symptoms. While our analyses suggested that O_3_ exposure modified the association between perceived support and asthma symptoms, rather than perceived support buffering against the effects of O_3_ exposure, after adjusting for covariates, the O_3_ × perceived support interaction was no longer statistically significant. While associations with intermittent vs. persistent symptoms were independent of SES (% poverty level) and daily prescription asthma medication use, the same pattern did not extend to reporting an asthma attack or a visit to the ER or urgent care because of asthma in the past year. Prior studies focused on stress in the family environment [4, 17, 18]; while the findings should be considered preliminary, this is the first study to examine whether outdoor air pollution exposure may strengthen the associations between perceived support in the home and symptom severity.

Two primary reasons for considering the reported findings preliminary are the differences between unadjusted and adjusted models and the cross-sectional study design. In adjusted models, poverty level was the only covariate systematically related to perceived support and was not systematically related to symptom frequency. In contrast, several covariates showed relatively strong associations with symptom frequency, age, Latino or African American ethnicity, and taking daily prescription control medication, with the latter two related to lower symptom frequency. Notably, other analyses of CHIS data found higher likelihood of daily asthma medication use among Latino and African American children [31, 33]. Thus, accounting for more severe symptoms in Latino and African American adolescents, as well as adolescents whose asthma severity necessitates daily asthma controller medications, may have reduced the O_3_ × perceived support effect.

These preliminary, cross-sectional data suggest several plausible interpretations that should be explored in future prospective longitudinal work. First, by increasing airway inflammation, pollution may also increase neural sensitivity to social experiences, which may further increase systemic and airway inflammation contributing to symptom severity. O_3_ interacts with molecules in the airway to stimulate innate inflammatory responses from lung macrophages and dendritic cells [2]. Experimental evidence shows that inducing inflammation through low-dose endotoxin injections has social cognitive effects [12], including heightened neural responses to pictures of close others in reward-related neural regions [13] and heightened neural responses to socially threatening pictures (fear faces) in threat-related neural regions like the amygdala [34]. Heightened amygdala reactivity to stressors may be related to heightened peripheral inflammation [35] and, in adult asthma patients, upregulated inflammatory signalling in the airway and increased airway inflammation [14]. Innate inflammatory response in the airway can then induce allergic inflammatory processes that lead to asthma exacerbations [36]. Furthermore, macrophages and dendritic cells are implicated in links between family conflict, exposure to chronic stressors, and a proinflammatory phenotype characterized by greater upregulation of genes regulated by the proinflammatory transcription factor nuclear factor-κB [23, 37] and lower sensitivity to anti-inflammatory glucocorticoids [9, 38].

Another possible interpretation is even though increased symptoms may occur because of high pollution exposure, a supportive home environment may alleviate the effects due to better adherence to medication and other treatment regimens. In cross-sectional data, greater family conflict or stress is related to lower adherence [39]; less is known about adult support and adolescent adherence in asthma. However, in this sample, greater perceived support was not related to reporting using daily prescription control medication.

The interpretations from this study should be considered in the context of limitations to inference and generalizability. This study focused on O_3_ rather than pollutants with high spatial variability like NO_x_ and particulate matter, where exposure modeling of multiple variables (e.g., traffic volume, wind speed, elevation, air monitoring data) enables estimating pollutant levels at a specific geographic location (i.e., respondent address) [4, 17, 18, 26]. O_3_ has less spatial variation compared to NOx, particularly in rural areas [2]. While air monitoring stations are less ideal for documenting personal exposures, estimates of risk associated with O_3_ exposure are generally similar across types of exposure assessment and spatial concentration of air monitors [2]. The population effect size for O_3_ and asthma symptoms is small (OR = 1.02 in children [3]), requiring thousands of children for sufficient power to observe a main effect of O_3_. While air monitoring data provided reliable estimates of average O_3_ exposures, acute O_3_ elevations may be more health-relevant than long-term exposures [2]. Finally, CHIS did not include measures of family conflict or stress (except for poverty level). Given that greater chronic stress, such as poverty, shows modest associations with more negative parenting including less perceived adolescent support [40, though for more nuanced data see 41], a critical direction for future work is examining whether interactions between support and pollution exposure are independent of proposed exacerbating effects of stress exposures [5–7]. Similarly, given the potential role of affective reactivity in proposed airway inflammation → social-cognitive neural circuitry → peripheral inflammation pathway, future work should determine whether interactions between social factors and pollution exposure are independent of (or explained by) emotion-related mechanisms.

Regarding generalizability, because of requirements related to living within 5 mi of an air monitoring station, the subsample of adolescents with asthma in CHIS is small, underpowered for detecting small effects, and may not be representative of adolescents with asthma in California or other regions. In our sample, greater perceived support showed a small to modest association with higher O_3_ levels, which may be explained by two observations in the literature. First, O_3_ concentrations are higher in suburban and rural areas that are downwind of air pollution sources, which are also higher income relative to urban areas [2]. Second, higher family SES is related to greater parent involvement in academics [42] and greater perceived parent social support during adolescence [43].

The observed findings are consistent with models that integrate psychosocial and pollution exposures to explain poor outcomes in at-risk populations [4–7, 9]. Future prospective research should include larger samples; more sophisticated family assessments, including indicators of affective sensitivity to the family environment; immune measures in the lung compartment; and personal exposures to pollution. Regarding translational implications, future work could test whether existing family interventions in asthma [e.g., 44] may show greater benefit in children living in high air pollution regions. Ultimately, behavioral pathways that increase adherence [22] or reduce pollution exposure via staying indoors or increasing exposure to green space [45] likely play a role in the interplay among family environments and air pollution. Finally, in this study, associations between perceived support at home and asthma symptoms were observed with exposures greater than 48 ppb, which is below the federal standard of 70 ppb. If future replications obtain similar results, the proportion of children with asthma that has health-relevant sensitivity to the home psychosocial environment may be considerable.

## Abbreviations

CARB: California Air Resources Board
CHIS: California Health Interview Survey
ER: Emergency room
mRNA: Messenger ribonucleic acid
NF-κB: Nuclear factor-κB
NO_2_: Nitrogen dioxide
NO_x_: Nitrogen dioxides
O_3_: Ozone
Ppb: Parts per billion

## Footnotes

1 The California *Healthy Kids Survey* was commissioned by the California Department of Education to assess student well-being in children age 10 and above (more information is available at https://calschls.org/about/the-surveys/).

2 According to the National Asthma Education and Prevention Program guidelines [29], intermittent asthma severity is defined in part as having daytime symptoms ≤ 2 days/week. Persistent asthma severity is defined as having daytime symptoms > 2 days/week. CHIS did not measure other components of severity (nighttime awakenings, use of short-term symptom control medications, daily interference, and lung function). The CHIS measure did not have fine-grained distinctions over a weekly timeframe but instead asked about a yearly timeframe (e.g., monthly, weekly). Thus, the 39.2% participants in the 5 mi sample (40.2%, 3 mi sample) who reported “weekly” and “daily” asthma could be considered as having persistent asthma according to published guidelines.
